# Supplementary material for: Multivalent interactions with CCR4–NOT and PABPC1 determine mRNA repression efficiency by tristetraprolin
Source: Nat Commun. 2025 Aug 13;16:7528. doi: 10.1038/s41467-025-62741-7 (PMC12350847; doi:10.1038/s41467-025-62741-7)
Supplement: Supplementary file 1 — Supplementary Information [file 41467_2025_62741_MOESM1_ESM.pdf]

SUPPLEMENTARY INFORMATION

Multivalent Interactions with CCR4-NOT and PABPC1 Determine mRNA Repression Efficiency by Tristetraprolin

Filip Pekovic[1], Wi S. Lai[2], Joshua Corbo[1], Stephanie N. Hicks[2], Keiko Luke[1], Perry J. Blackshear[2,3], Eugene Valkov[1]

[1]National Cancer Institute, National Institutes of Health, Frederick, MD 21702, USA.
[2]National Institute of Environmental Health Sciences, National Institutes of Health, Research Triangle Park, Durham, NC 27709, USA.
[3]Departments of Medicine and Biochemistry, Duke University Medical Center, Durham, NC 27710, USA.

Supplementary Table 1. Proteins investigated in this study.

Protein	UniProt ID	No. of residues	Molecular mass (kDa)	Synonyms
**Tristetraprolin**	P26651	326	34	TTP, ZFP36, TIS11A
**ZFP36L1**	Q07352	338	36.3	TIS11B, BRF1
**ZFP36L2**	P47974	494	51.1	TIS11D, BRF2
**NOT1**	A5YKK6-1	2376	266.9	CNOT1
**NOT2**	Q9NZN8-1	540	59.7	CNOT2
**NOT3**	O75175	753	81.9	CNOT3
**NOT9**	Q92600-1	299	33.6	CAF40, CNOT9, RCD-1
**NOT6**	Q9ULM6	557	63.3	CCR4a, CNOT6
**NOT7**	Q9UIV1-1	285	32.7	CAF1, CNOT7
**NOT10**	Q9H9A5-1	744	82.3	CNOT10
**NOT11**	Q9UKZ1	510	55.2	CNOT11, C2ORF29
**PABPC1**	P11940-1	636	70.7	PABP
**14-3-3 β**	P31946-1	246	28.1	Protein 1054, KCIP-1
**14-3-3 η**	Q04917	246	28.2	Protein AS1

Supplementary Table 2. Plasmids used in this study

**The following constructs were all cloned into the pnYC plasmid (for bacterial expression) with a TEV-cleavable, N-terminal MBP-tag, and a C-terminal StrepII-tag. If not otherwise stated, cDNA was PCR amplified and Gibson assembled into pnYC cut open with the indicated restrictions sites.**			
**Protein**	**Residues**	**Restriction sites**	**Comments**
**TTP**	1-326	NdeI/XhoI	
**ZFP36L1**	1-338	NdeI/XhoI	
**ZFP36L2**	1-494	NdeI/BamHI	
**TTP(C124R)**	1-326	-	Site-specific mutagenesis of TTP
**TTP(∆CNBD)**	1-313	NdeI/XhoI	TTP(∆CNBD) is synonymous to TTP(1-313)
**TTP(N)**	1-101	NdeI/XhoI	
**TTP(TZF)**	102-169	NdeI/XhoI	
**TTP(C)**	170-326	NdeI/XhoI	
**TTP(N-TZF)**	1-169	NdeI/XhoI	
**TTP(TZF-C)**	102-326	NdeI/XhoI	TTP(TZF-C) is synonymous to TTP(102-326)
**TTP(1-274)**	1-274	NdeI/AvrII	

**Protein**	**Residues**	**Restriction sites**	**Comments**
**TTP(1-248)**	1-248	NdeI/AvrII	
**TTP(1-223)**	1-223	NdeI/AvrII	
**TTP(102-313)**	102-313	NdeI/XhoI	
**TTP(102-248)**	102-248	NdeI/AvrII	
**TTP(102-223)**	102-223	NdeI/AvrII	
**TTP(∆224-248)**	1-326 with ∆224-248	NdeI/XbaI	Multi-fragment Gibson assembly
**TTP(∆249-274)**	1-326 with ∆249-274	NdeI/XbaI	Multi-fragment Gibson assembly
**TTP(∆224-274)**	1-326 with ∆224-274	NdeI/XbaI	Multi-fragment Gibson assembly
**TTP(275-326)**	275-326	NdeI/AvrII	
**TTP(275-313)**	275-313	NdeI/AvrII	
**TTP(1xW/A)**	1-326 with W32A	KpnI/XbaI	OE-PCR mutagenesis
**TTP(2xW/A)**	1-326 with W32A/W38A	KpnI/XbaI	OE-PCR mutagenesis with TTP(1xW/A) as template for PCR
**TTP(3xW/A)**	1-326 with W32A/W38A/W69A	KpnI/XbaI	OE-PCR mutagenesis with TTP (2xW/A) as template for PCR
**TTP(4xW/A)**	1-326 with W32A/W38A/W69A/W262A	KpnI/XbaI	OE-PCR mutagenesis with TTP (3xW/A) as template for PCR

**The following plasmids were all cloned into the pLIB plasmid (for baculoviral expression) with a TEV-cleavable, N-terminal MBP-tag, and a C-terminal StrepII-tag.**			
**Protein**	**Residues**	**Restriction sites**	**Comments**
**TTP**	1-326	BamHI/XbaI	TTP DNA cut out of the pnYC-TTP plasmid with BglII/XbaI
**ZFP36L1**	1-338	BamHI/XbaI	ZFP36L1 DNA was cut out of the pnYC-ZFP36L1 plasmid with BglII/XbaI
**ZFP36L2**	1-494	BamHI/XbaI	ZFP36L2 DNA was cut out of the pnYC-ZFP36L2 plasmid with BglII/XbaI
**TTP(TZF)**	102-169	BamHI/XbaI	TTP(TZF) DNA was cut out of the pnYC-TTP(TZF) plasmid with BglII/XbaI
**TTP(N-TZF)**	1-169	BamHI/XbaI	TTP(N-TZF) DNA was cut out of the pnYC-TTP(N-TZF) plasmid with BglII/XbaI
**TTP(TZF-C)**	102-326	BamHI/XbaI	TTP(TZF-C) DNA was cut out of the pnYC-TTP(TZF-C) plasmid with BglII/XbaI
**TTP(1-274)**	1-274	BamHI/XbaI	TTP(1-274) DNA was cut out of the pnYC-TTP(1-274) plasmid with BglII/AvrII
**TTP(1-248)**	1-248	BamHI/XbaI	TTP(1-248) DNA was cut out of the pnYC-TTP(1-248) plasmid with BglII/AvrII
**TTP(1-223)**	1-223	BamHI/XbaI	TTP(1-248) DNA was cut out of the pnYC-TTP(1-248) plasmid with BglII/AvrII
**TTP(249-326)**	249-326	NdeI/XbaI	TTP(249-326) was Gibson assembled into pLIB-TTP(1-326) cut with NdeI/XbaI
**TTP(275-326)**	275-326	BamHI/XbaI	TTP(275-326) DNA was cut out of the pnYC-TTP(275-326) plasmid with BglII/AvrII
**TTP(275-313)**	275-313	BamHI/XbaI	TTP(275-313) DNA was cut out of the pnYC-TTP(275-313) plasmid with BglII/AvrII

The following constructs were generated for transfection of HEK-293 cells.

Construct	Encoded	Plasmid	Restriction sites	Protein tag	Comments
**CMV.MBP.TTP.Strep**	TTP(1-326)	pBS+(CMV.BGH3')	Asp718/XhoI	N-term MBP, C-term StrepII	No TEV site between MBP and TTP
**mIL-3.HiBiT**	mouse IL-3 cDNA	pSK-	SalI/SstI	C-term HiBiT	
**CMV.sec.mCherry**	mCherry	pBS+(CMV.BGH3')	Asp718/NotI	N-term IL-6 signal peptide	
**CMV.EGFP.BGH3'**	EGFP	pBS+(CMV.BGH3')	HindIII/NOT1		
**CMV.nes.EGFP**	EGFP	pBS+(CMV.EGFP.BGH3')	combine with previous vector	N-term TTP nuclear export signal	
**CMV.TTP.EGFP**	TTP(1-326)	pBS+(CMV.EGFP.BGH3')	Asp718/AgeI	C-term EGFP	
**CMV.TTP(C124R).EGFP**	TTP(1-326), with C124R	pBS+(CMV.TTP.EGFP)		C-term EGFP	OE-PCR mutagenesis with CMV-TTP.EGFP as template for PCR
**CMV.TTP(1-313).EGFP**	TTP(1-313)	pBS+(CMV.TTP.EGFP)	SmaI/AgeI	C-term EGFP	
**CMV.TTP(1-274).EGFP**	TTP(1-274)	pBS+(CMV.TTP.EGFP)	SmaI/AgeI	C-term EGFP	

**CMV.TTP(1-248).EGFP**	TTP(1-248)	pBS+(CMV.TTP.EGFP)	SmaI/AgeI	C-term EGFP	

Construct	Encoded	Plasmid	Restriction sites	Protein tag	Comments
**CMV.TTP(1-206).EGFP**	TTP(1-206)	pBS+(CMV.TTP.EGFP)	SmaI/AgeI	C-term EGFP	
**CMV.TTP(3xW/A).EGFP**	TTP(1-326) with W32A/W38A/W69A	pBS+(CMV.TTP.EGFP)		C-term EGFP	OE-PCR mutagenesis with CMV-TTP.EGFP as template for PCR
**CMV.TTP(∆249-274).EGFP**	TTP(1-326) with ∆249-274	pBS+(CMV.TTP.EGFP)		C-term EGFP	OE-PCR mutagenesis with CMV-TTP.EGFP as template for PCR
**CMV.TTP(∆CNBD+∆249-274).EGFP**	TTP(1-313) with ∆249-274	pBS+(CMV.TTP.EGFP)		C-term EGFP	OE-PCR mutagenesis with CMV-TTP.EGFP as template for PCR
**CMV.TTP(∆CNBD+3xW/A).EGFP**	TTP(1-313) with W32A/W38A/W69A	pBS+(CMV.TTP.EGFP)		C-term EGFP	OE-PCR mutagenesis with CMV-TTP.EGFP as template for PCR
**pC1-TTP-Strep**	TTP(1-326)	mEGFP-C1	NheI/BamHI	C-term StrepII	

**Construct**	**Encoded**	**Plasmid**	**Restriction sites**	**Protein tag**	**Comments**
**pC1-TTP(C124R)-Strep**	TTP(1-326) with C124R	mEGFP-C1	NheI/BamHI	C-term StrepII	
**pC1-TTP(4xW/A)-Strep**	TTP(1-326) with W32A/W38A/W69A/W262A	mEGFP-C1	NheI/BamHI	C-term StrepII	
**pC1-TTP(ΔCNBD)-Strep**	TTP(1-313)	mEGFP-C1	NheI/BamHI	C-term StrepII	
**pC1-TTP(4xW/A+ΔCNBD)-Strep**	TTP(1-313) with W32A/W38A/W69A/W262A	mEGFP-C1	NheI/BamHI	C-term StrepII	
**pC1-TTP(1-248)-Strep**	TTP(1-248)	mEGFP-C1	NheI/BamHI	C-term StrepII	
**pC1-TTP(1-223)-Strep**	TTP(1-223)	mEGFP-C1	NheI/BamHI	C-term StrepII	
**pC1-TTP(8xS/A)-Strep**	TTP(1-326) with S273A/S276A/S279A/S286A/S287A/S289A/S290A/S296A	mEGFP-C1	NheI/BamHI	C-term StrepII	
**pC1-PH-mCherry**	PH-mCherry	mEGFP-C1	NheI/EcoRI		
**pC1-OST4-mCherry**	PH-mCherry	mEGFP-C1	NheI/EcoRI		

**pC1-PH-mCherry-TTP**	TTP(1-326)	pC1-PH-mCherry	BamHI/EcoRI	N-term PH-mCherry	

Construct	Encoded	Plasmid	Restriction sites	Protein tag	Comments
**pC1-OST4-mCherry-NANOS3(1-117)**	NANOS3(1-117)	pC1-OST4-mCherry	BamHI/EcoRI	N-term OST4-mCherry	
**pC1-mEGFP-NOT1(SHD)**	NOT1(1847-2361)	mEGFP-C1	HindIII/BamHI	N-term mEGFP	

**The following constructs were cloned into the pETM11 plasmid (for bacterial expression), with a TEV-cleavable, N-terminal His_6-tag. If not otherwise stated, cDNA was PCR amplified and Gibson assembled into plasmid cut open with the indicated restrictions sites.**				
**Protein**	**Residues**	**Plasmid**	**Restriction sites**	**Comments**
**PABPC1**	1-636	pETM11	NcoI/NotI	
**PABPC1(M1)**	1-636 with K174E/R176E/R179E	pETM11		Site directed mutagenesis on pETM11-PABPC1
**PABPC1(M2)**	1-636 with K268E/K269E/R272E	pETM11		Site directed mutagenesis on pETM11-PABPC1
**PABPC1(M1 +M2)**	1-636 with K174E/R176E/R179E /K268E/K269E/ R272E	pETM11		Site directed mutagenesis on pETM-PABPC1(M1)

The following constructs were either cloned into the pnYC or pnEK plasmids (for bacterial expression). If not otherwise stated, cDNA was PCR amplified and Gibson assembled into plasmid cut open with the indicated restrictions sites.

Protein	Residues	Plasmid	Restriction sites	Affinity tag	Comments
**PABPC1(∆MLLE)**	1-541	pnYC	NdeI	N-term His_6-MBP (TEV cleavable)	
**PABPC1(RRM1-4)**	1-368	pnYC	NdeI	N-term His_6-MBP (TEV cleavable)	
**PABPC1(∆RRM1-4)**	369-636	pnYC	NdeI	N-term His_6-MBP (TEV cleavable)	
**PABPC1(RRM1-2)**	1-190	pnEK	NdeI	N-term His_6 (TEV cleavable)	
**PABPC1(RRM2-3)**	91-287	pnEK	NdeI	N-term His_6 (TEV cleavable)	
**PABPC1(RRM3-4)**	191-392	pnYC	BamHI/NdeI	N-term His_6 (HRV-3C cleavable)	Additional α-helix downstream of RRM4 (residues 369-392)

The following constructs were cloned into the pET28a(+) plasmid. Synthetic DNA was assembled into plasmid cut open with the indicated restriction sites.

Protein	Restriction sites	Affinity tag	Comments
**14-3-3 β**	BamHI/HindIII	N-term His_6-T7	
**14-3-3 η**	BamHI/HindIII	N-term His_6-T7	

Supplementary Table 3. RNA substrates used in this study.

Name	Sequence	Comments
**ARE-WT-RNA**	UUAUUUAUUAAAAAAAAAAAAAAAAAAAA	9 nt long ARE upstream of 20 nt long poly(A). 5' end carries an Atto 532 fluorescent dye.
**ARE-MUT-RNA**	UU<u>C</u>UUU<u>C</u>UUAAAAAAAAAAAAAAAAAAAA	9 nt long ARE was mutated at the two underlined sites. RNA has a 20 nt long poly(A) tail. 5' end carries a 6-FAM fluorescent dye.
**ARE-WT-RNA (A30)**	UUAUUUAUUAAAAAAAAAAAAAAAAAAAAAAAAAAAAAA	9 nt long ARE upstream of 30 nt long poly(A). 5' end carries a 6-FAM fluorescent dye.
**ARE-WT-RNA (A60)**	UUAUUUAUUAAAAAAAAAAAAAAAAAAAAAAAAAAAAAAAAAAAAAAAAAAAAAAAAAAAAAAAAAAAA	9 nt long ARE upstream of 60 nt long poly(A). 5' end carries a 6-FAM fluorescent dye.
**A30**	AAAAAAAAAAAAAAAAAAAAAAAAAAAAAA	Used for competition experiments.
**C30**	CCCCCCCCCCCCCCCCCCCCCCCCCCCCCC	Used for competition experiments.

Supplementary Table 4. qPCR primer.

Name	Sequence	Origin
**Forw_Rplp0**	CGTCCTCGTGGAAGTGACAT	This study
**Rev_Rplp0**	ATCTGCTTGGAGCCCACATT	This study
**Forw_IER3**	CTTGGCCTTACACTCCGCTC	This study
**Rev_IER3**	CTGGCGCCGGACCACT	This study
**Forw_CXCR4**	ACGCCACCAACAGTCAGAG	PrimerBank ID: 56790928c3
**Rev_CXCR4**	AGTCGGGAATAGTCAGCAGGA	PrimerBank ID: 56790928c3
**Forw_PIM3**	GACTTCGACGGCACCCGA	This study
**Rev_PIM3**	ATCGTAGAGAAGCACGCCCA	This study

SUPPLEMENTARY METHODS

Electrophoretic mobility shift assays (EMSA)

EMSA (electrophoretic mobility shift assay) binding reactions contained 50 nM substrate RNA and 400 nM TTP proteins or 200 nM pZFP36L2. Reactions were carried out for 15 min at 37 °C in a buffer containing 20 mM PIPES pH 6.8, 10 mM KCl, 40 mM NaCl, 2 mM $Mg(OAc)_2$, 3% v/v Ficoll 400, and 0.05% v/v NP-40. The RNA-protein complexes were analyzed by electrophoresis on a nondenaturing polyacrylamide gel in 0.5x TBE buffer, pH 8.3, at 10 V cm^{-1}. Fluorescent RNA substrates were visualized by scanning with a Typhoon RGB biomolecular imager (Cytiva).

For competition experiments with 14-3-3 proteins (**Supplementary Fig. 7g**), both pZFP36L2 and 14-3-3 were preincubated for 45 minutes before addition of RNA substrate to allow protein complex formation.

Confocal fluorescence microscopy

$1x10^5$ HEK-293T cells were seeded per well of a 12 well glass bottom plate (Cellvis, P12-1.5H-N). After 24 h the cells were transfected using FuGENE HD transfection reagent (Promega) and 0.5 µg per CMV-driven vector. Live-cell imaging was conducted ca. 24 h post-transfection using a 63X, oil, 1.4 NA, 0.19 mm objective on a Zeiss LSM 780 confocal microscope (resolution 1024x1024) in a 37 °C incubation chamber. ZEN 2012 SP5 (version 14.0.20.201) was used for image acquisition. Images were processed and quantified with Fiji[1] (version 2.16.0/1.54p).

Western Blotting

Protein samples were resolved on 4-12 % SurePAGE Bis-Tris precast gels (GenScript) and transferred to methanol-activated PVDF membranes using the Trans-blot Turbo transfer system (Bio-Rad). Membranes were blocked with 5% blotting-grade blocker (non-fat dry milk, Bio-Rad) in PBST (0.1% Tween 20) for 1 h at room temperature and gentle rocking. Membranes were rinsed with PBST before incubating with the indicated primary antibodies (1:1000 dilution in PBST) overnight at 4 °C with gentle shaking. Afterwards, membranes were quickly rinsed three times with PBST before doing three ca. 5 min washes with 10 ml PBST. Secondary antibody was diluted 1:2000 in PBST and added to the membrane for 1 h at room temperature under constant rocking. Membranes were washed as described for the primary antibody. Blots were developed for ca. 1 min using the SuperSignal West Pico Plus ECL substrate (Thermo Fisher) and SuperSignal West Femto Maximum Sensitivity Substrate (Thermo Fisher). Images were captured with an ImageQuant 800 (Amersham).

Antibodies

Primary antibodies: anti-StrepII (Abcam, ab307676, clone EPR28119-43, monoclonal); anti-Hsp90 (Proteintech, 11405-1-AP, polyclonal); anti-PABPC1 (Abcam, ab21060, Lot: GR3218800-1, polyclonal). Secondary antibody: horseradish peroxidase(HRP)-linked anti-rabbit IgG (Cell Signaling Technology, 7074P2, Lot: 29)

Mass spectrometry

In-solution trypsin digestion: 2 µL of sample (2-5 µg; with n = 1) was treated with 25 µL reducing solution and 25 µL alkylating solution provided with the EasyPep digestion kit (Thermo, A45733), 50 µL 100 mM HEPES pH 8, and 10µL of 40 ng/µL Trypsin/LysC. Samples were incubated at 37 °C for 1 h before being treated with 100 µL of 1% formic acid and cleaned using an EasyPep 96-well plate provided with the kit. Peptides were eluted in 200µL of elution buffer and dried. TTP overexpressed and purified from *E. coli* served as a phosphorylation negative control.

In-gel trypsin digestion: A band corresponding to the protein of interest for each sample was destained in 50% acetonitrile and 25 mM NH_4HCO_3 followed by a reduction with 10 mM TCEP for 60 min. TCEP was removed and 100 µL of 20 ng/µL Trypsin/LysC was added to each gel band and incubated for 1 hr on ice. The Trypsin/LysC solution was removed and 200 µL of 50 mM HEPES pH 8 was added to each gel band and incubated at 37°C overnight. The supernatant was removed and 100 µL of 1% formic acid was added before using the EasyPep 96-well plate for further purification. Peptides were eluted in 200 µL of elution buffer and dried.

Other proteolytic digestion**:** For each sample, 2 µL (2-5 µg) was placed in 4 separate tubes labeled A-D. Each tube was treated with 7 µL of reducing solution and 7µL of alkylating solution provided with the EasyPep digestion kit and incubated at room temperature for 60 min. For each sample, tubes A-C were treated with 20 µL of 100 mM HEPES pH 8 followed by addition of 2 µL of 0.1 µg/µL of the following proteases: A) Chymotrypsin, B) GluC, and C) Thermolysin. Digestions were carried out at 37 °C for 1 h before addition of 60 µL of 0.15% TFA to acidify and stop the reaction. Tube D was treated with 20 µL of 100 mM HCl (pH ~1) followed by addition of 2 µL of 0.05 µg/µL ProAlanase (Promega). Tube D was incubated at 37 °C for 15 min, then heated at 95 °C for 10 min to inactivate the enzyme before the addition of 62 µL of 100 mM HEPES pH 8. For each sample, the 4 digestions were combined and treated with 200 µL of 1% formaldehyde and cleaned using EasyPep 96-well plate provided with the kit. Peptides were eluted in 200 µL of elution buffer and dried.

LC/MS/MS analysis: For all samples, each of the three sets of dried peptides were resuspended in 50 µL 0.1% formaldehyde and 10 µL were analyzed by LC/MS/MS using a Q-Exactive HF equipped with an Easy nLC 1200 (Thermo). Peptides were loaded onto a trap column (Acclaim™ PepMap™ 100 C18

HPLC Column, 3µm, 75µm I.D., 2cm, Thermo 164946) in-line with the analytical column (EasySpray C18 HPLC Column, 2µm, 75µm I.D., 25cm, Thermo ES902). The gradient pump was operated at a flow rate of 300 nL/min and each run used a linear LC gradient of 5-7% B for 1 min, 7-25% B for 20 min, 25-40% B for 10 min, 40-98% B for 1 min, then holding at 98% B for 10 min. For MS acquisition the spray voltage was 1750 V and ion transfer temperature was 250 °C. MS1 scans were acquired in the Orbitrap with a resolution of 60,000, AGC of $3x10^6$ ions, max injection time of 120 ms, mass range of 400-1600 m/z. MS2 scans were acquired in the Orbitrap for the top 20 ions in each MS1 scan with a resolution of 15,000, AGC of $2x10^5$, max injection time of 50 ms, HCD energy of 27%, isolation width of 1.4 Da, intensity threshold of $2x10^4$ and charges 2-6 for MS2 selection. Monoisotopic Precursor selection was enabled, and dynamic exclusion was set to a count of 1 for 15 sec.

Database Search and processing: All MS files were searched against the Uniprot Human database from August 2023 in Proteome Discoverer 2.4 using the Sequest node and Fixed Value PSM for FDR calculation. The three injections for each sample were searched independently then combined after the search. The in-solution and in-gel trypsin/LysC digests were searched using a full tryptic digest while the combined 4-protease digest was searched using 4 different Sequest nodes, one for each of the 4 proteases with full cleavage (Chymotrypsin = FWYL, GluC = DE, ProAlanase = PA, Thermolysin = FAIMLV). Each search had 2 max missed cleavages, minimum peptide length of 5 amino acids, maximum peptide length of 40 amino acids, an MS1 mass tolerance of 10 ppm, MS2 mass tolerance of 0.02 Da, variable oxidation on methionine (+15.995 Da), variable phosphorylation on serine, threonine, and tyrosine (+79.966), fixed modification of carbamidomethyl on cysteine (+57.021), and variable protein N-terminus modifications of acetyl (+42.011), Met-loss (-131.040), and Met-loss+acetyl (-89.030).

Supplementary Figure 1

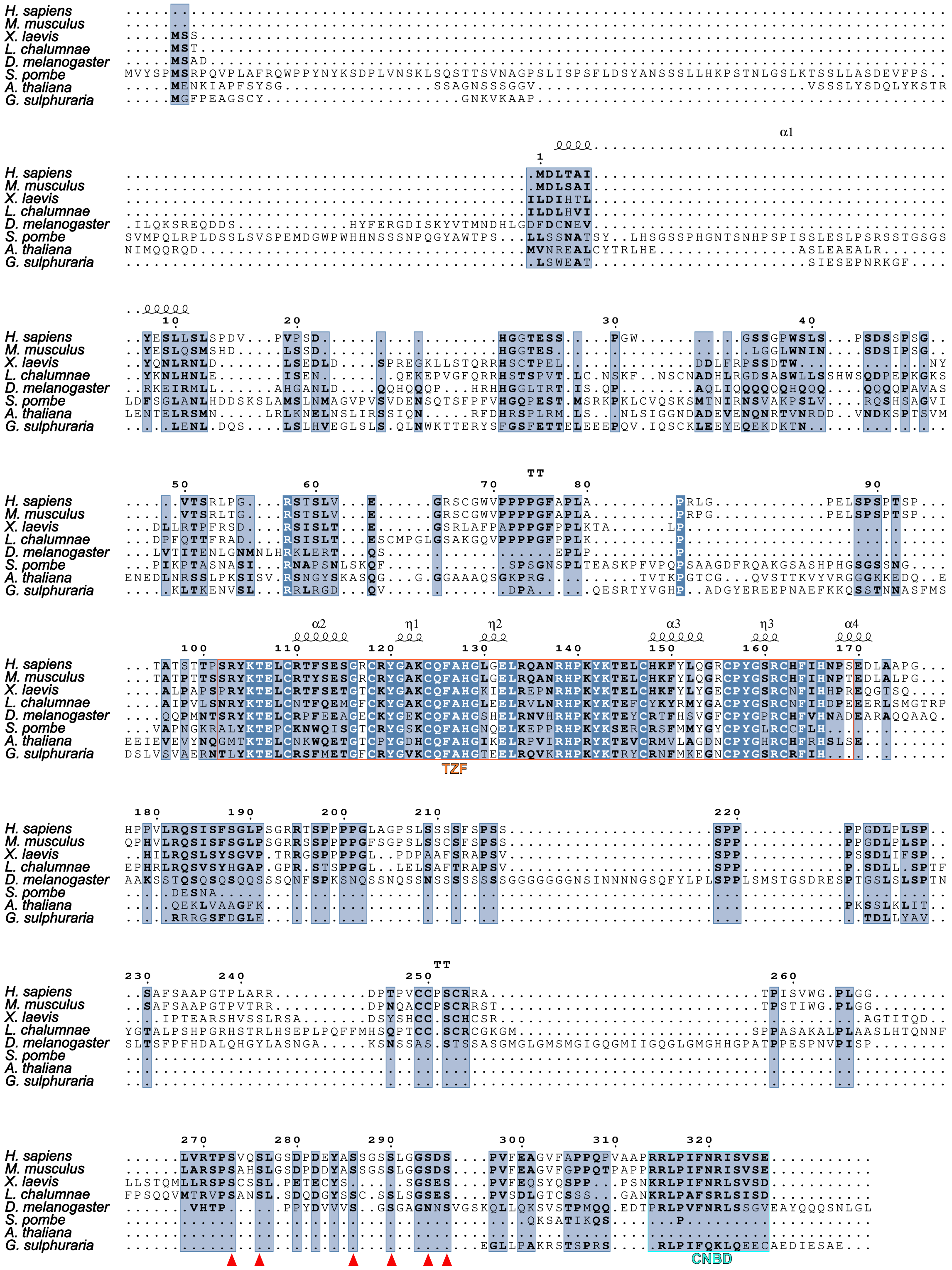

Supplementary Figure 1. Known CCR4-NOT interaction sites are not conserved among all eukaryotes.

Sequences of TTP orthologs from eukaryotes were aligned with the MAFFT program in SnapGene v7.2.1. The alignment was further processed in ESPript v3.0[2]. Residues that are fully conserved or highly conserved, are highlighted in dark blue with white letter font and light blue with black font, respectively. Similar residues (physico-chemical properties) are emphasized with bold font. The regions encompassing the TZF (orange frame) and CNBD (turquoise frame) are labeled. Conserved serine residues involved in PABPC1 interaction are indicated by red triangles.The secondary structure on top of the aligned sequences was based on an AlphaFold2 prediction of human TTP. UniProt identifiers are given for each sequence: *H. sapiens* (Human; P26651), *M. musculus* (Mouse; P22893), *X. laevis* (African clawed frog; Q9W671), *L. chalumnae* (Coelacanth; H3B8B8), *D. melanogaster* (Fruit fly; P47980), *S. pombe* (Fission yeast; P47979), *A. thaliana* (Mouse-ear cress; Q9C9F5-1), *G. sulphuraria* (Red alga; M2VUA0).

Supplementary Figure 2

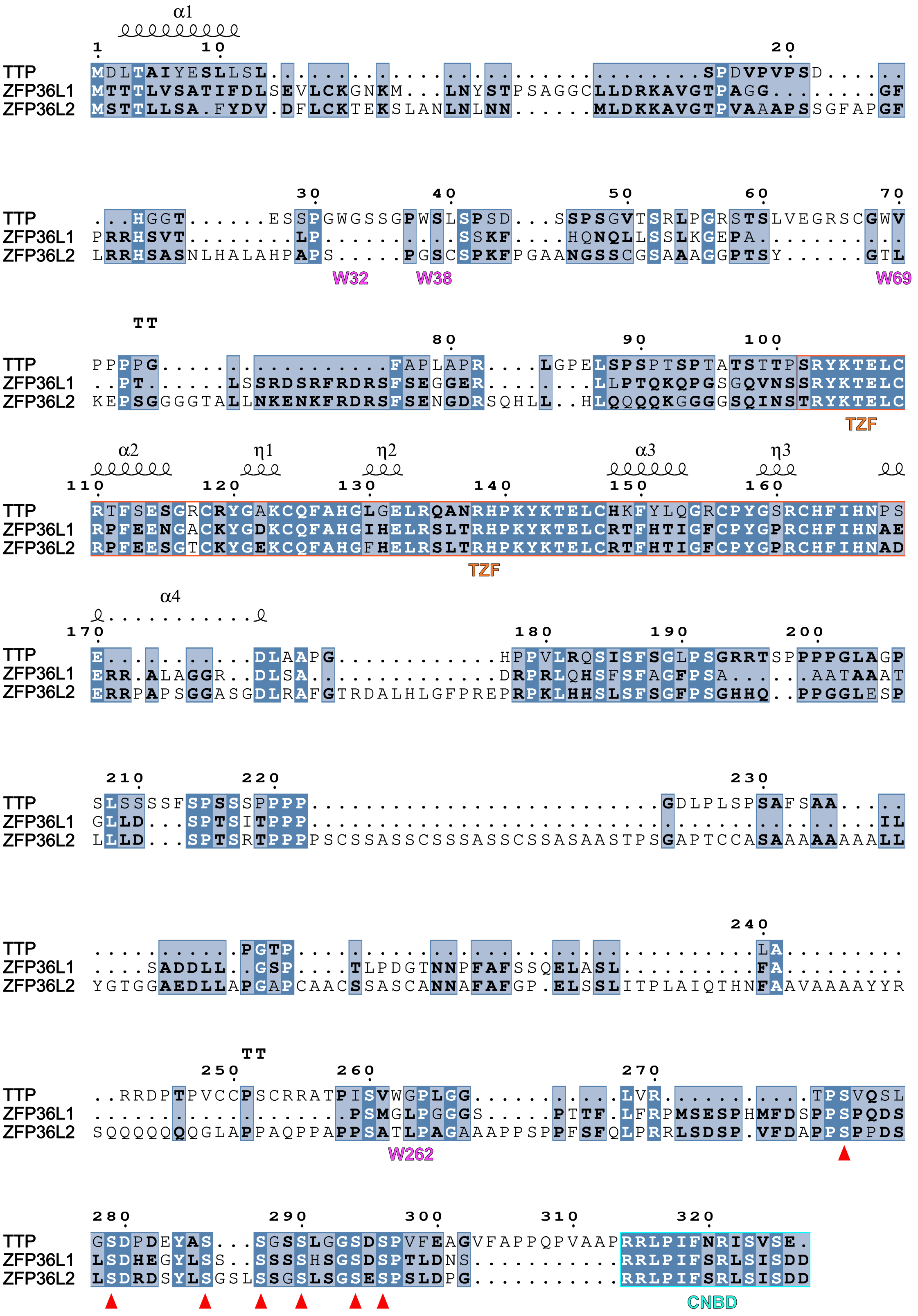

Supplementary Figure 2. Tryptophan residues are not conserved among human TTP family members.

Sequences of human TTP, ZFP36L1, and ZFP36L2 were aligned with the MAFFT program in SnapGene v7.2.1. The alignment was further processed in ESPript v3.0[2]. Residues that are fully conserved or highly conserved, are highlighted in dark blue with white letter font and light blue with black font, respectively. Similar residues (physico-chemical properties) are emphasized with bold font. The secondary structure on top of the aligned sequences was based on an AlphaFold2 prediction of human TTP. The regions encompassing the TZF (orange frame), CNBD (turquoise frame), and tryptophan residues in TTP are labeled. Conserved serine residues involved in PABPC1 interaction are indicated by red triangles. UniProt identifiers are given for each sequence: TTP (P26651), ZFP36L1 (Q07352), ZFP36L2 (P47974).

Supplementary Figure 3

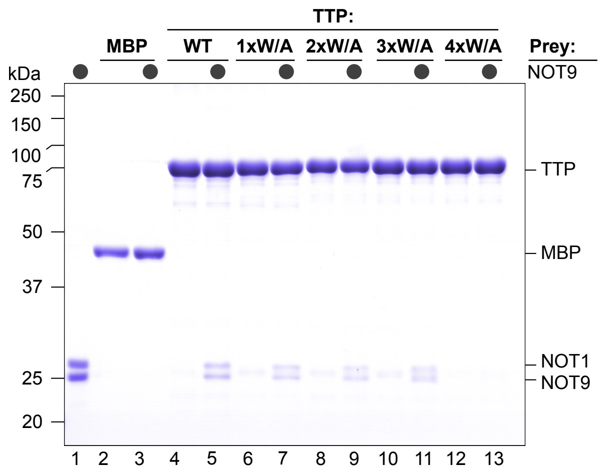

Supplementary Figure 3. At least one tryptophan residue is necessary for interaction with NOT9.

Pull-down assays between the NOT9 module (black circles) and TTP (WT, wild-type) or TTP variants with tryptophan residues mutated to alanine. WT, wild-type TTP; 1×W/A, W32A; 2×W/A, W32A/W38A; 3×W/A, W32A/W38A/W69A; 4×W/A, W32A/W38A/W69A/W262A. MBP was included as a negative control. Here and in all other figures, proteins were resolved by SDS-PAGE and visualized by Coomassie staining. Representative gel from three independent experiments is shown.

Supplementary Figure 4

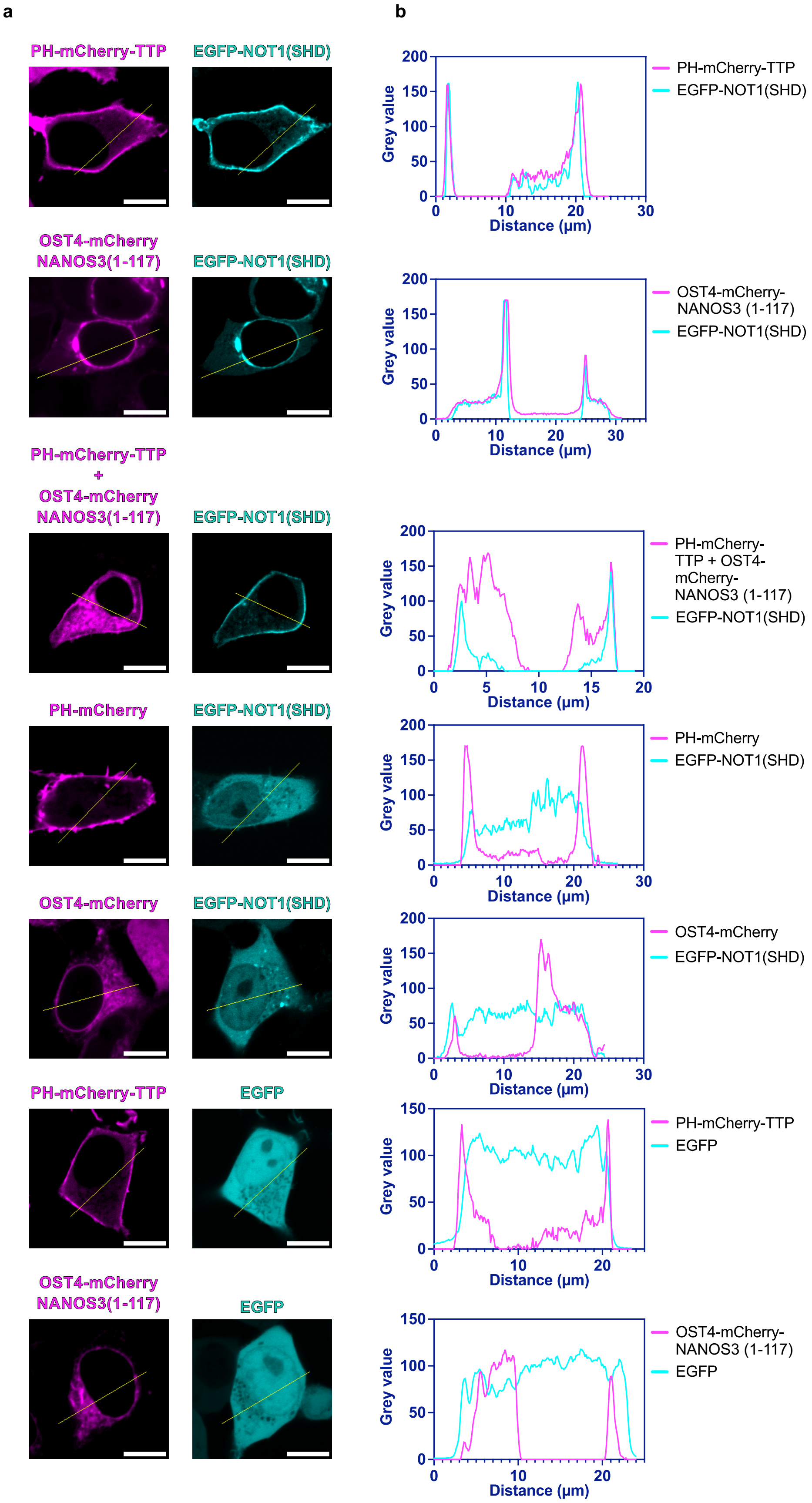

Supplementary Figure 4. Interaction between TTP and NOT1(SHD) of the NOT module in HEK-293T cells.

(a) Relocalization assays showing the colocalization between plasma membrane localized PH-mCherry-TTP, ER localized OST4-mCherry-NANOS3(1-117), and EGFP-NOT1(SHD). Scale bar, 10 µm. Profile lines (yellow) were drawn for quantification of grey values shown in (b). Representative of three biological replicates.

Supplementary Figure 5

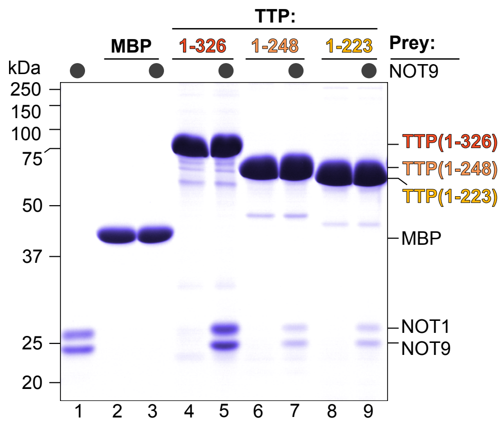

Supplementary Figure 5. Truncation of the C-terminal IDR decreases interaction with NOT9.

Pull-down assays assessing the ability of full-length TTP (1-326) or C-terminal IDR truncations (as indicated) to bind the NOT9 module (black circles). MBP was included as a negative control. Representative gel from three independent experiments is shown.

Supplementary Figure 6

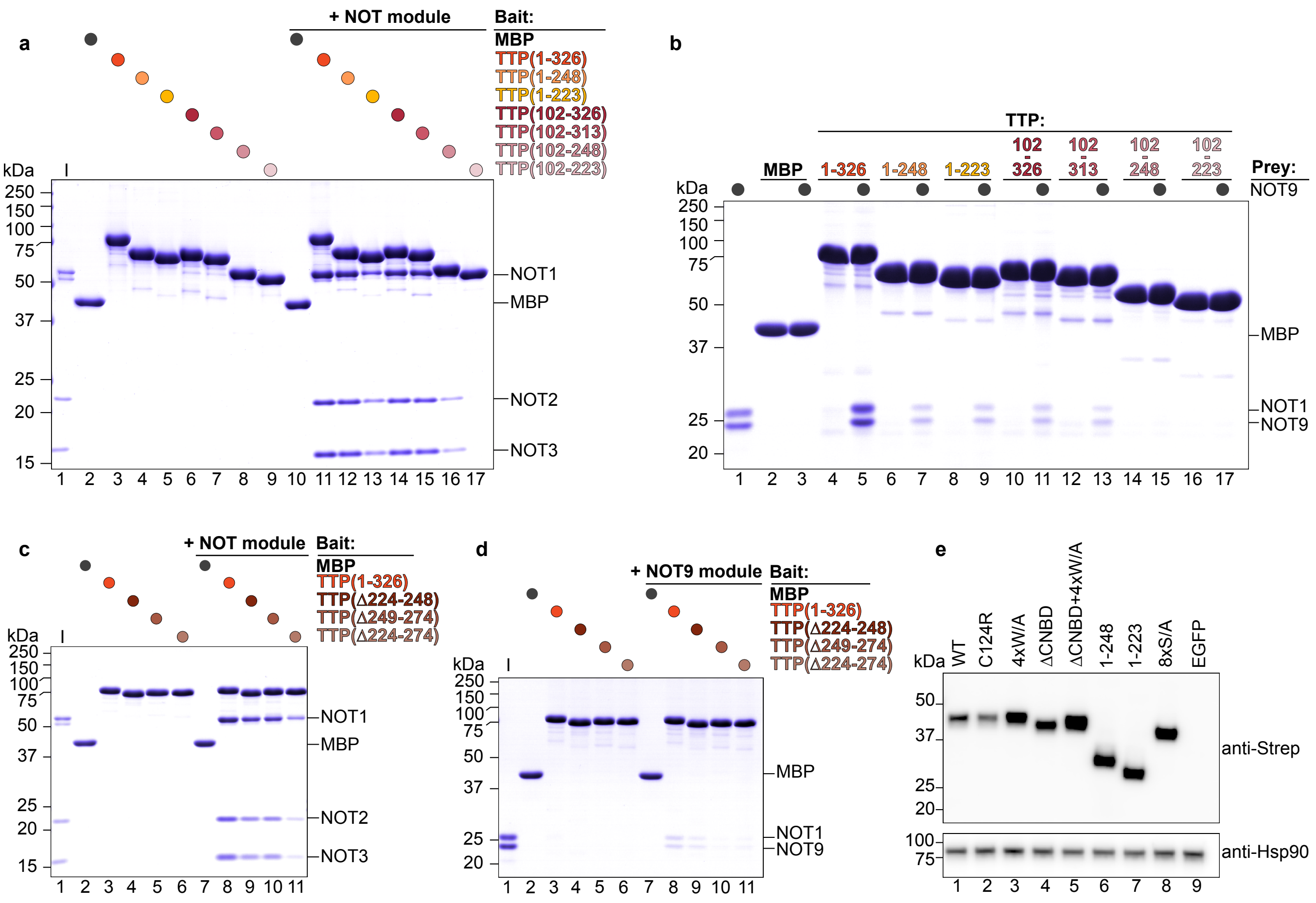

Supplementary Figure 6. Multiple regions in the C-terminal IDR are important for interaction with CCR4-NOT.

(a,b) Pull-down assays analyzing the interaction of TTP (colored circles in panel a) with C-terminal truncations, either alone or combined with deletion of the N-terminal IDR, with the NOT module (panel a) or the NOT9 module (panel b, black circles). MBP was included as a negative control (black circles in panel a). Representative gel from three independent experiments is shown.

(c,d) Pull-down assays testing deletion variants of the identified repressor region within TTP (colored circles) for their ability to interact with the NOT module (panel c) or the NOT9 module (panel d). MBP was included as a negative control (black circles). Representative gel from three independent experiments is shown.

(e) Immunoblot showing the transient overexpression of TTP variants in HEK-293T cells. C-terminal StrepII-tag on TTP was used for detection. Hsp90 was used as a reference protein. Representative of five biological replicates.

Supplementary Figure 7

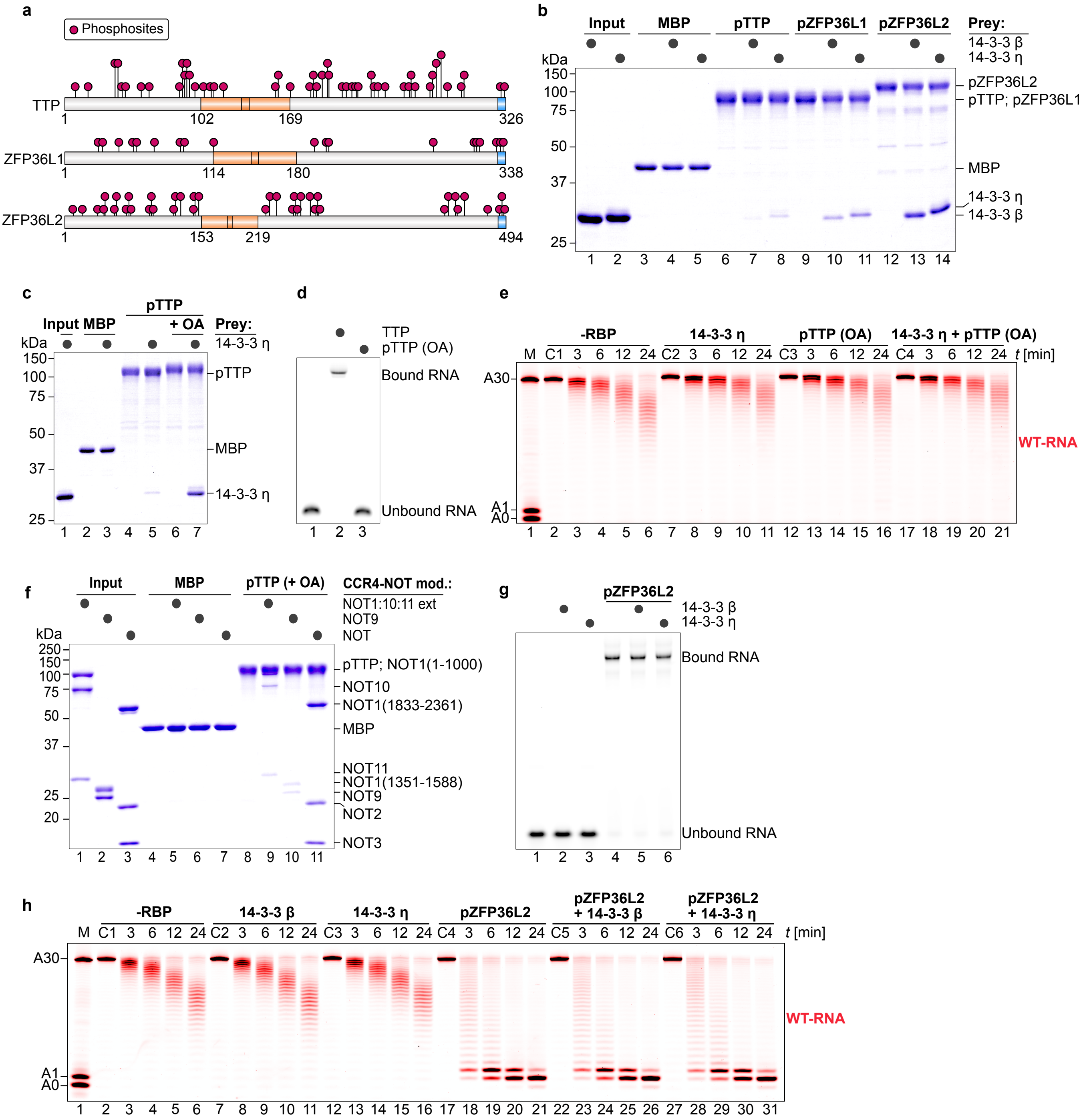

Supplementary Figure 7. Hyperphosphorylation of TTP directly blocks RNA-binding but facilitates interaction with 14-3-3 proteins.

(a) Diagram illustrating previously reported phosphorylation sites (red circles) in TTP and its family members, based on the PhosphoSitePlus database[3].

(b) Pull-down assays showing the interactions between phosphorylated TTP (pTTP) and its human family members, pZFP36L1 and pZFP36L2, with 14-3-3 proteins (black circles). All TTP family members were expressed in Sf21 insect cells. MBP was included as a negative control. Representative gel from three independent experiments is shown.

(c) Pull-down assays demonstrating the effect of hyperphosphorylation of TTP on its association with 14-3-3 η. Immobilized TTP was overexpressed in HEK-293T cells either under okadaic acid (OA) exposure or not. Representative gel from three independent experiments is shown.

(d) EMSA showing the loss of interaction between TTP and the ARE-containing substrate RNA due to okadaic acid (OA) induced hyperphosphorylation of TTP. Substrate RNA (50 nM) was incubated with 400 nM TTP protein. Here and in all other figures, RNAs were resolved by native PAGE and visualized by fluorescence detection. Representative gel from three independent experiments is shown.

(e) Deadenylation assay comparing the stimulation of deadenylation by pTTP (OA) and preincubation of pTTP (OA) with 14-3-3 η. pTTP (OA) was added at 100 nM, in four-fold excess over CCR4-NOT (25 nM) and twofold excess over substrate RNAs (50 nM). 14-3-3 η (2000 nM) was added in twentyfold excess over pTTP (OA). Controls included reactions containing either RNA alone (C1) or RNA with 14-3-3 η (C2), pTTP (OA) (C3), or both proteins (C4). Here and in all other figures, RNAs were resolved by urea-PAGE and visualized by fluorescence detection. The first lane here and in all following deadenylation assay gels indicates the size marker corresponding to the number of adenosines present. Representative gel from three independent experiments is shown.

(f) Pull-down assays testing the interactions between hyperphosphorylated TTP due to okadaic acid (OA) exposure and CCR4-NOT modules (black circles). MBP was included as a negative control. Representative gel from three independent experiments is shown.

(g) EMSA analyzing the effect of pZFP36L2 interaction with 14-3-3 proteins on its binding to an ARE-containing substrate RNA. pZFP36L2 (200 nM) was preincubated with a tenfold excess of 14-3-3 β or η

(2000 nM) before addition of the substrate RNA (50 nM). Representative gel from three independent experiments is shown.

(h) Deadenylation assay testing the effect of a preformed pZFP36L2:14-3-3 complex on targeted deadenylation. pZFP36L2 was added at 100 nM, in fourfold excess over CCR4-NOT (25 nM) and twofold excess over substrate RNAs (50 nM). 14-3-3 β or η (2000 nM) were added in twentyfold excess over pZFP36L2. Controls included reactions containing either RNA alone (C1) or RNA with 14-3-3 β (C2) or η (C3), pZFP36L2 (C4), pZFP36L2 with 14-3-3 β (C5) or η (C6). Representative gel from three independent experiments is shown.

Supplementary Figure 8

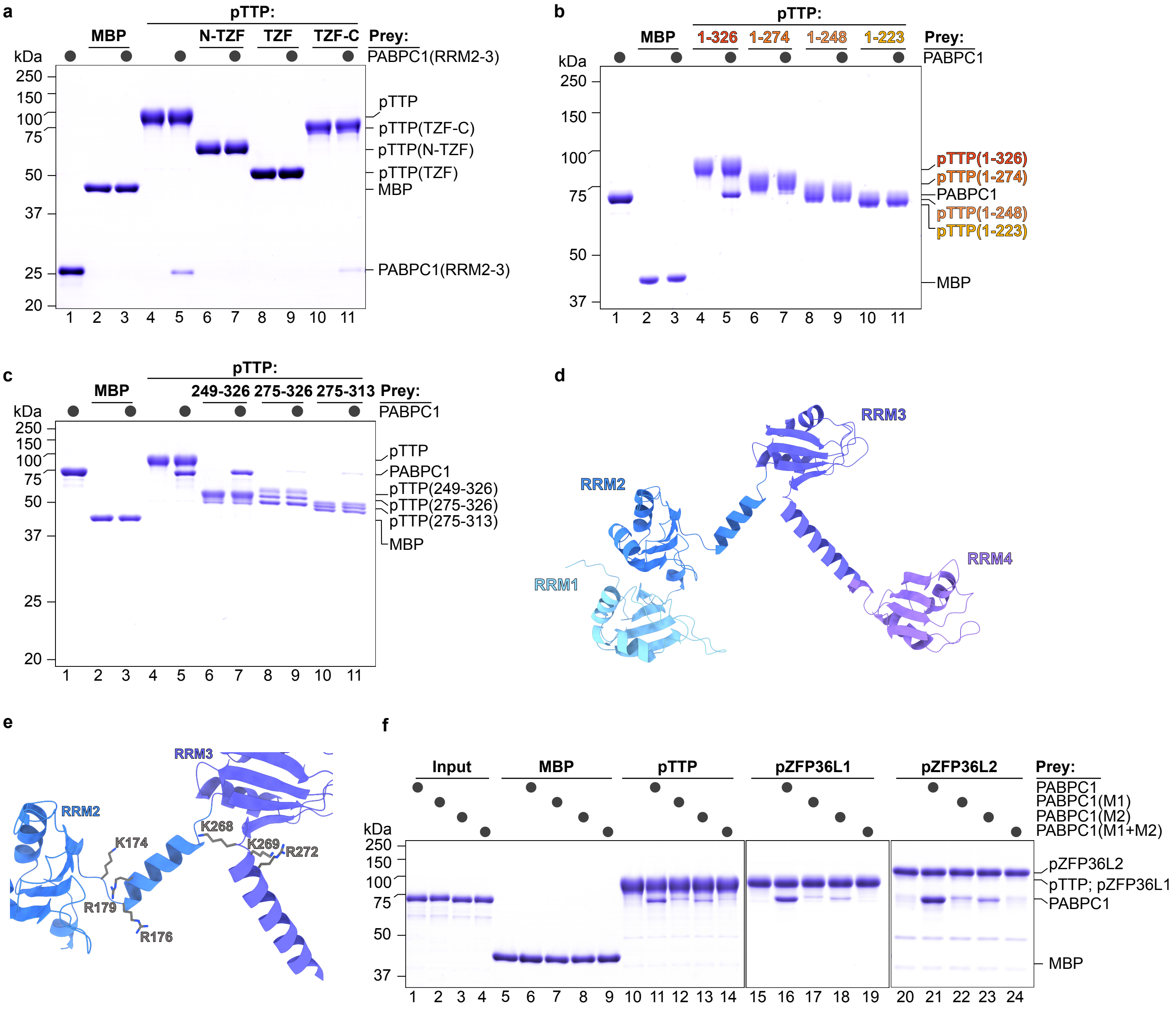

Supplementary Figure 8. A short region in the C-terminal IDR of pTTP interacts with PABPC1.

(a) Pull-down assays examining pTTP or pTTP fragments for interaction with the RRM2–3 region (black circles) of PABPC1. MBP was included as a negative control. Representative gel from three independent experiments is shown.

(b) Pull-down assays with truncations of the C-terminal IDR of pTTP to identify a specific region crucial for interaction with PABPC1 (black circles). MBP was included as a negative control. Representative gel from three independent experiments is shown.

(c) Pull-down assays testing if the identified region within the C-terminal IDR can independently interact with PABPC1 (black circles). MBP was included as a negative control. Representative gel from three independent experiments is shown.

(d) AlphaFold2 prediction of the RRMs of PABPC1, showing the conserved α-helical linkers between RRM2 and RRM4.

(e) Visualization of positively charged, surface-exposed residues in the α-helical linkers between RRM2 and RRM4 of PABPC1; potential interaction sites for phosphorylated TTP orthologs and its family members.

(f) Pull-down assays examining the effect of charge-reversal mutations in the α-helical linkers between RRM2 and RRM4 of PABPC1 (M1: K174E, R176E, R179E; M2: K268E, K269E, R272E) on interaction with pTTP, pZFP36L1 and pZFP36L2. Representative gel from three independent experiments is shown.

Supplementary Figure 9

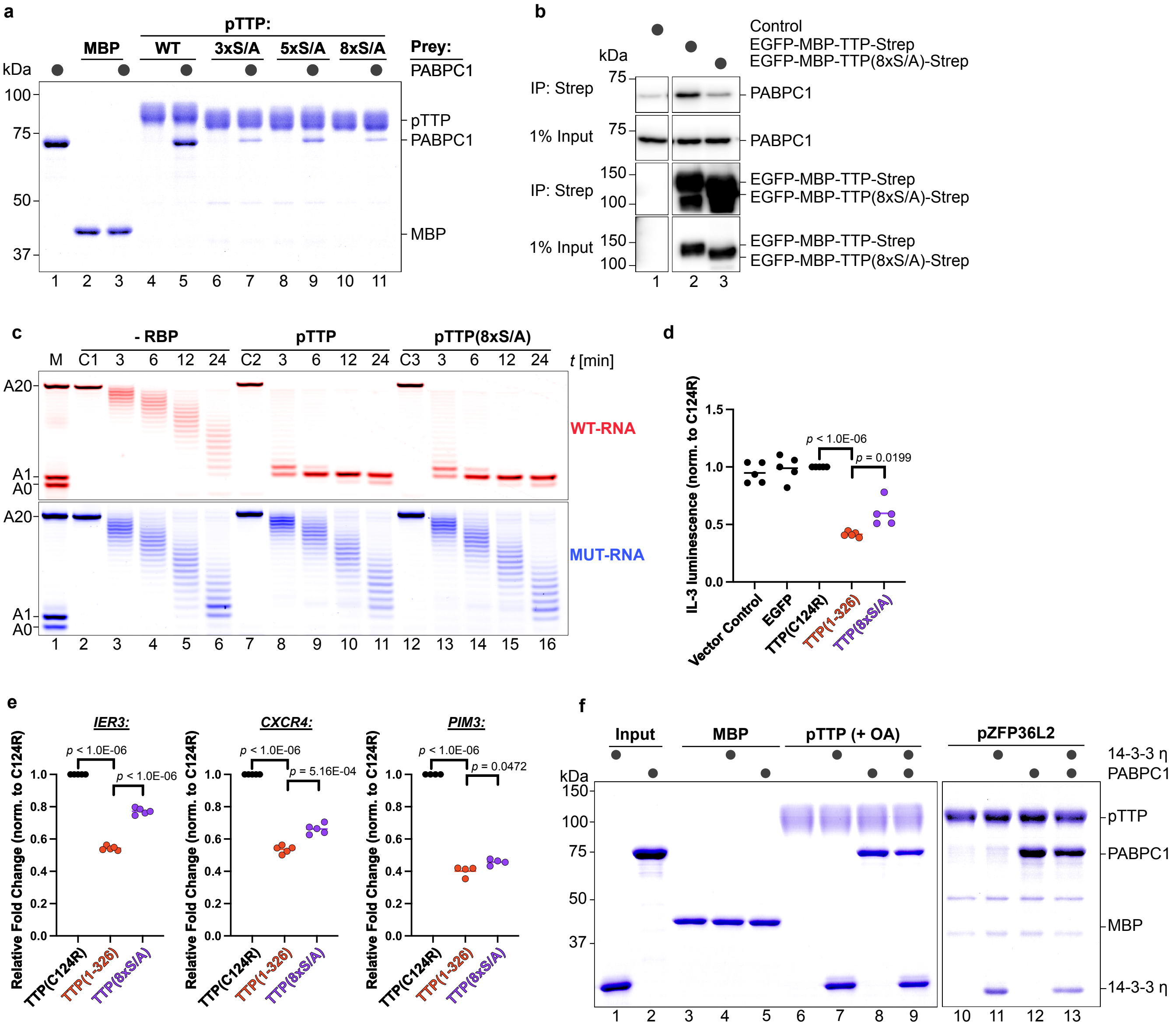

Supplementary Figure 9. A short stretch of conserved serine residues in TTP is necessary for interaction with PABPC1.

(a) Pull-down assays showing reduced interaction between pTTP and PABPC1 (black circles) upon mutation of conserved serine residues in TTP to alanine. The following serine residues were mutated to alanine: S273, S276, S279 (3xS/A); S286, S287, S289, S290, S296 (5xS/A); all of the aforementioned (8xS/A). MBP was included as a negative control. Representative gel from three independent experiments is shown.

(b) Pull-down assays testing the interaction between endogenous PABPC1 with transiently overexpressed TTP or 8xS/A mutant. Cells transfected with EGFP were included as a negative control. Shown is a western blot with antibodies probing for PABPC1 or the C-terminal StrepII-tag on TTP proteins. Representative of three biological replicates.

(c) Deadenylation assay examining the effect of the 8xS/A mutations in TTP on its stimulation of deadenylation. pTTP or 8xS/A mutant was added at 100 nM, in twofold excess over CCR4-NOT (50 nM) and substrate RNAs (50 nM). Controls included reactions containing either RNA alone (C1) or RNA with pTTP (C2), or 8xS/A mutant (C3). Representative gel from three independent experiments is shown.

(d) The effect of the 8xS/A mutations in TTP on the repression of IL-3 protein production was measured via secreted IL-3 luminescence. Each data point represents one biological replicate (n = 5).

(e) RT-qPCR of endogenous *IER3*, *CXCR4*, and *PIM3* in HEK-293T cells transduced with CMV-driven vectors expressing wild-type TTP (1-326) or 8xS/A mutant. Each data point represents one biological replicate (n = 5 for *IER3* and *CXCR4*, n = 4 for *PIM3*). Statistical significance in (d,e) was determined using a two-tailed Student's *t*-test followed by Holm-Šidák multiple comparison test and shown as exact *p* values.

(f) Pull-down assays to assess the ability of PABPC1 to interact simultaneously with 14-3-3 η and hyperphosphorylated pTTP due to okadaic acid (OA) exposure or pZFP36L2. Complexes between pTTP or pZFP36L2 (each 250 pmol) and 14-3-3 η (1000 pmol) were formed before addition of PABPC1 (500 pmol). MBP was included as a negative control. Representative gel from three independent experiments is shown.

Supplementary References

1. Schindelin, J. *et al.* Fiji - an Open Source platform for biological image analysis. *Nat. Methods* **9**, 676–682 (2012).
2. Robert, X. & Gouet, P. Deciphering key features in protein structures with the new ENDscript server. *Nucleic Acids Res.* **42**, W320-4 (2014).
3. Hornbeck, P. V. *et al.* PhosphoSitePlus, 2014: mutations, PTMs and recalibrations. *Nucleic Acids Res.* **43**, D512-20 (2015).
