# Supplementary material for: Multivalent interactions with CCR4–NOT and PABPC1 determine mRNA repression efficiency by tristetraprolin
Source: Nat Commun. 2025 Aug 13;16:7528. doi: 10.1038/s41467-025-62741-7 (PMC12350847; doi:10.1038/s41467-025-62741-7)
Supplement: Supplementary file 2 — Description of Additional Supplementary Files [file 41467_2025_62741_MOESM2_ESM.docx]

**Supplementary Data:**

**File Name:** Supplementary Data 1

**Description:** MS-analysis of post-translational modifications in TTP family members.
