# Supplementary material for: Multivalent interactions with CCR4–NOT and PABPC1 determine mRNA repression efficiency by tristetraprolin
Source: Nat Commun. 2025 Aug 13;16:7528. doi: 10.1038/s41467-025-62741-7 (PMC12350847; doi:10.1038/s41467-025-62741-7)
Supplement: Supplementary file 4 — Reporting Summary [file 41467_2025_62741_MOESM4_ESM.pdf]

nature portfolio

Corresponding author(s): Eugene Valkov

Last updated by author(s): Jul 2, 2025

Reporting Summary

Nature Portfolio wishes to improve the reproducibility of the work that we publish. This form provides structure for consistency and transparency in reporting. For further information on Nature Portfolio policies, see our Editorial Policies and the Editorial Policy Checklist.

Statistics

For all statistical analyses, confirm that the following items are present in the figure legend, table legend, main text, or Methods section.

n/a	Confirmed	
☐	☒	The exact sample size (*n*) for each experimental group/condition, given as a discrete number and unit of measurement
☐	☒	A statement on whether measurements were taken from distinct samples or whether the same sample was measured repeatedly
☐	☒	The statistical test(s) used AND whether they are one- or two-sided<br>*Only common tests should be described solely by name; describe more complex techniques in the Methods section.*
☒	☐	A description of all covariates tested
☐	☒	A description of any assumptions or corrections, such as tests of normality and adjustment for multiple comparisons
☒	☐	A full description of the statistical parameters including central tendency (e.g. means) or other basic estimates (e.g. regression coefficient) AND variation (e.g. standard deviation) or associated estimates of uncertainty (e.g. confidence intervals)
☐	☒	For null hypothesis testing, the test statistic (e.g. *F*, *t*, *r*) with confidence intervals, effect sizes, degrees of freedom and *P* value noted<br>*Give P values as exact values whenever suitable.*
☒	☐	For Bayesian analysis, information on the choice of priors and Markov chain Monte Carlo settings
☒	☐	For hierarchical and complex designs, identification of the appropriate level for tests and full reporting of outcomes
☒	☐	Estimates of effect sizes (e.g. Cohen's *d*, Pearson's *r*), indicating how they were calculated

Our web collection on statistics for biologists contains articles on many of the points above.

Software and code

Policy information about availability of computer code

Data collection	Detection of fluoresence of RNA gels: Amersham Typhoon control software v1.1.0.7 (GE Healthcare)<br>Detection of fluorescence and luminescence in IL-3 co-transfection assays: Magellan Pro v7.5 software (Tecan).<br>Live-cell imaging was conducted using a 63X, oil, 1.4 NA, 0.19 mm objective on a Zeiss LSM 780 confocal microscope (resolution 1024x1024).<br>ZEN 2012 SP5 (version 14.0.20.201) was used for image acquisition during microscopy.<br>LC/MS/MS was done using a Q-Exactive HF equipped with an Easy nLC 1200 (Thermo).<br>qPCR experiments were carried out with a CFX Opus 96 (Bio-Rad).
Data analysis	Data anlysis was conducted with GraphPad Prism v10.3.1.<br>Microscopy images were analyzed and processed with FiJi software (version 2.16.0/1.54p).<br>Mass spectrometry files were analyzed with Proteome Discoverer (version 2.4).<br>qPCR experiments were run using CFX Maestro (version 5.0.021.0616).

For manuscripts utilizing custom algorithms or software that are central to the research but not yet described in published literature, software must be made available to editors and reviewers. We strongly encourage code deposition in a community repository (e.g. GitHub). See the Nature Portfolio guidelines for submitting code & software for further information.

Data

Policy information about availability of data

All manuscripts must include a data availability statement. This statement should provide the following information, where applicable:

- Accession codes, unique identifiers, or web links for publicly available datasets
- A description of any restrictions on data availability
- For clinical datasets or third party data, please ensure that the statement adheres to our policy

All data supporting the findings of this study are available within the paper and its Supplementary Information. The raw data associated with the gel images, and deadenylation assays, cell-based assays can be found in the Source Data file. Specific data p values and statistical analysis are also included within the Source Data file. The raw and processed mass spectrometry proteomics data have been deposited to the MassIVE repository under accession MSV000098386 [doi:10.25345/C5FQ9QJ40]. The raw microscopy image data generated in this study has been deposited in the figshare database under the accession code https://doi.org/10.6084/m9.figshare.29451836.

Research involving human participants, their data, or biological material

Policy information about studies with human participants or human data. See also policy information about sex, gender (identity/presentation), and sexual orientation and race, ethnicity and racism.

Reporting on sex and gender	n/a
Reporting on race, ethnicity, or other socially relevant groupings	n/a
Population characteristics	n/a
Recruitment	n/a
Ethics oversight	n/a

Note that full information on the approval of the study protocol must also be provided in the manuscript.

Field-specific reporting

Please select the one below that is the best fit for your research. If you are not sure, read the appropriate sections before making your selection.

☒ Life sciences ☐ Behavioural & social sciences ☐ Ecological, evolutionary & environmental sciences

For a reference copy of the document with all sections, see nature.com/documents/nr-reporting-summary-flat.pdf

Life sciences study design

All studies must disclose on these points even when the disclosure is negative.

Sample size	No sample-size calculations was performed. Sample size were chosen based on similar experiments in the literature and considered sufficient based on the reproducibility of the experiments. All replicates show same result.
Data exclusions	Dead or apoptotic HEK-293T cells were excluded from microscopy.
Replication	All assays are supported by at least three technical replicates and two biological replicates. All attempts at replication were successful.
Randomization	No randomization was necessary because this study is based on fully-controlled biochemical experiments.
Blinding	The investigators were not blinded for experiments and outcome assessment. The investigator who did the cell transfection also did the microscopy imaging and analysis. Blinding was not done because there was a clear, objective measurement (relocalization to distinct cellular compartments) that can be obtained without subjective interpretation.

Reporting for specific materials, systems and methods

We require information from authors about some types of materials, experimental systems and methods used in many studies. Here, indicate whether each material, system or method listed is relevant to your study. If you are not sure if a list item applies to your research, read the appropriate section before selecting a response.

Materials & experimental systems

n/a	Involved in the study
☐	☒ Antibodies
☐	☒ Eukaryotic cell lines
☒	☐ Palaeontology and archaeology
☒	☐ Animals and other organisms
☒	☐ Clinical data
☒	☐ Dual use research of concern
☒	☐ Plants

Methods

n/a	Involved in the study
☒	☐ ChIP-seq
☒	☐ Flow cytometry
☒	☐ MRI-based neuroimaging

Antibodies

Antibodies used	anti-StrepII (Abcam, ab307676, clone:EPR28119-43, monoclonal, 1:1000 dilution in PBST); anti-Hsp90 (Proteintech, 11405-1-AP, polyclonal, 1:1000 dilution in PBST); anti-PABPC1 (Abcam, ab21060, Lot:GR3218800-1, polyclonal, 1:1000 dilution in PBST); horseradish peroxidase(HRP)-linked anti-rabbit IgG (Cell Signaling Technology, 7074P2, Lot:29, 1:2000 dilution in PBST)
Validation	All antibodies were either purchased from Abcam, Proteintech, or Cell Signaling Technology. Primary antibodies were validated by manufacturer:<br><br>anti-StrepII (Abcam, ab307676, clone:EPR28119-43) - Specificity and sensitivity confirmed in IHC with multi-tissue microarray (TMA) validation; validation information in datasheet (page 1): https://www.abcam.com/en-us/products/primary-antibodies/strep-tag-ii-antibody-epr28119-43-ab307676?srsltid=AfmBOor-RuZfwr5WrBf9ouES-Re6XVqjVgz0hKwb1oib1IPA496fZ3ES#tab=datasheet<br><br>anti-PAPBC1 (Abcam, ab21060, Lot:GR3218800-1, polyclonal) - sensitivity was validated by western blotting of multiple eukaryotic tissues and cell types, page 6 in product datasheet https://www.abcam.com/en-us/products/primary-antibodies/pabp-antibody-ab21060?srsltid=AfmBOooltLHnOZ51KDkcEGYk2z8kW_IC7syLNMFLAcy_wLFKTHED0KKT#tab=datasheet;<br><br>anti-Hsp90 (Proteintech, 11405-1-AP, polyclonal) - validation via western blot analysis among different cell types/tissues, and in a knock-down/knock-out background, third image in validation gallery https://www.ptglab.com/products/HSP90AB1-Antibody-11405-1-AP.htm?srsltid=AfmBOopNLKWVJ9L2p4ACKOv6GepxawA6fbNGAanIRQHieCzdtGR0hr07.<br><br>HRP-linked anti-rabbit IgG (Cell Signaling Technology, 7074P2) is a secondary antibody purchased from Cell Signaling Technology.

Eukaryotic cell lines

Policy information about cell lines and Sex and Gender in Research

Cell line source(s)	Sf21 insect cells for was obtained from Thermo Fisher Scientific (catalog #11497013). HEK-293 cells were obtained from ATCC (CRL-1573). HEK-293T cells were obtained from DSMZ (ACC 635)
Authentication	Sf21 cells were not authenticated. HEK-293 and HEK-293T cells growth characteristics and morphology were consistent with descriptions and images provided by ATCC and DSMZ.
Mycoplasma contamination	HEK-293T cells were tested negative for mycoplasma contamination. Sf21 and HEK-293 cell lines were not tested for mycoplasma contamination.
Commonly misidentified lines (See ICLAC register)	No commonly misidentified cell lines were used in the study.

Plants

Seed stocks	*Report on the source of all seed stocks or other plant material used. If applicable, state the seed stock centre and catalogue number. If plant specimens were collected from the field, describe the collection location, date and sampling procedures.*
Novel plant genotypes	*Describe the methods by which all novel plant genotypes were produced. This includes those generated by transgenic approaches, gene editing, chemical/radiation-based mutagenesis and hybridization. For transgenic lines, describe the transformation method, the number of independent lines analyzed and the generation upon which experiments were performed. For gene-edited lines, describe the editor used, the endogenous sequence targeted for editing, the targeting guide RNA sequence (if applicable) and how the editor was applied.*
Authentication	*Describe any authentication procedures for each seed stock used or novel genotype generated. Describe any experiments used to assess the effect of a mutation and, where applicable, how potential secondary effects (e.g. second site T-DNA insertions, mosiacism, off-target gene editing) were examined.*
